# Latent Risk Intrahepatic Cholangiocarcinoma Susceptible to Adjuvant Treatment After Resection: A Clinical Deep Learning Approach

**DOI:** 10.3389/fonc.2020.00143

**Published:** 2020-02-19

**Authors:** Seogsong Jeong, Yang Ge, Jing Chen, Qiang Gao, Guijuan Luo, Bo Zheng, Meng Sha, Feng Shen, Qingbao Cheng, Chengjun Sui, Jingfeng Liu, Hongyang Wang, Qiang Xia, Lei Chen

**Affiliations:** ^1^Department of Liver Surgery, School of Medicine, Renji Hospital, Shanghai Jiao Tong University, Shanghai, China; ^2^International Cooperation Laboratory on Signal Transduction, Eastern Hepatobiliary Surgery Institute, Second Military Medical University, Shanghai, China; ^3^School of Public Health, Shanghai Jiao Tong University School of Medicine, Shanghai, China; ^4^Department of Liver Surgery and Transplantation, Liver Cancer Institute, Zhongshan Hospital, and Key Laboratory of Carcinogenesis and Cancer Invasion (Ministry of Education), Fudan University, Shanghai, China; ^5^Eastern Hepatobiliary Surgery Hospital, Second Military Medical University, Shanghai, China; ^6^Biliary Tract Department I, Eastern Hepatobiliary Surgery Hospital, Second Military Medical University, Shanghai, China; ^7^Department of Special Medical Care and Liver Transplantation, Eastern Hepatobiliary Surgery Hospital, Second Military Medical University, Shanghai, China; ^8^The United Innovation of Mengchao Hepatobiliary Technology Key Laboratory of Fujian Province, Mengchao Hepatobiliary Hospital of Fujian Medical University, Fuzhou, China; ^9^Department of Oncology, Shanghai Cancer Center and Institutes of Biomedical Sciences, Shanghai Medical College, Fudan University, Shanghai, China

**Keywords:** biliary malignancy, artificial intelligence, prognostic factor, prediction model, primary liver cancer

## Abstract

**Background:** Artificial Intelligence (AI) frameworks have emerged as a novel approach in medicine. However, information regarding its applicability and effectiveness in a clinical prognostic factor setting remains unclear.

**Methods:** The AI framework was derived from a pooled dataset of intrahepatic cholangiocarcinoma (ICC) patients from three clinical centers (*n* = 1,421) by applying the TensorFlow deep learning algorithm to Cox-indicated pathologic (four), serologic (six), and etiologic (two) factors; this algorithm was validated using a dataset of ICC patients from an independent clinical center (*n* = 234). The model was compared to the commonly used staging system (American Joint Committee on Cancer; AJCC) and methodology (Cox regression) by evaluating the brier score (BS), integrated discrimination improvement (IDI), net reclassification improvement (NRI), and area under curve (AUC) values.

**Results:** The framework (BS, 0.17; AUC, 0.78) was found to be more accurate than the AJCC stage (BS, 0.48; AUC, 0.60; IDI, 0.29; NRI, 11.85; *P* < 0.001) and the Cox model (BS, 0.49; AUC, 0.70; IDI, 0.46; NRI, 46.11; *P* < 0.001). Furthermore, hazard ratios greater than three were identified in both overall survival (HR; 3.190; 95% confidence interval [CI], 2.150–4.733; *P* < 0.001) and disease-free survival (HR, 3.559; 95% CI, 2.500–5.067; *P* < 0.001) between latent risk and stable groups in validation. In addition, the latent risk subgroup was found to be significantly benefited from adjuvant treatment (HR, 0.459; 95% CI, 0.360–0.586; *P* < 0.001).

**Conclusions:** The AI framework seems promising in the prognostic estimation and stratification of susceptible individuals for adjuvant treatment in patients with ICC after resection. Future prospective validations are needed for the framework to be applied in clinical practice.

## Introduction

Artificial Intelligence (AI) is a field of computer science in which machines mimic, recognize, and learn cognitive functions of the human mind and make empirical predictions using task-specific algorithms (1, 2). It is natural for the human mind to get confused when trying to process a lot of information simultaneously, and this necessitates an auxiliary process. This need has led to the application of AI in clinical medicine (3). AI has been applied to develop a diagnostic tool using image-based deep learning (DL), and the resulting performance was close to that of humans (4). However, no study has applied an AI framework to identify patients prone to the latent risk of recurrence even after curative treatment.

Intrahepatic cholangiocarcinoma (ICC) is a highly aggressive primary epithelial malignancy arising from the liver, and it has witnessed rising interests over the years due to rapid increase in its incidence and the resulting mortality rate (5, 6). Usually, ICC is diagnosed at an advanced stage, sporadically and without an explicit etiologic factor, thereby limiting curative approaches (7). Surgery with curative intent is the current standard of care, providing the opportunity for long-term survival (8). However, due to frequent recurrence of ICC, less than half of the post-surgery patients have been reported to survive for more than 5 years (9).

Despite clinical challenges, the growing understanding of ICC, led by increased investigations, is providing new insight into heterogeneity, pathogenesis, and therapeutic strategies with regard to the disease (10–12). Locally advanced ICC is no longer a contraindication for transplantation, and adjuvant treatments are now implemented more frequently worldwide, suggesting that it is important to identify the prognostic subtype for all treatments (13, 14). However, prognostic subtypes that support the selection of therapeutic modality remain limited, especially for recurrent ICC.

Owing to the exponential increase in the number of ICC studies, prognosis of the disease is witnessing development (15). Various prognosis-predictive systems with biological, pathological, demographic, clinical, and imaging characteristics have been developed (16, 17). However, such systems could not be implemented widely due to their inaccuracy and discriminations against them. To address this issue, we have developed a DL computational framework for ICC. The framework was tested in subgroups of patients who received prophylactic adjuvant transarterial chemoembolization (PAT), post-recurrent chemotherapy (PRC), post-recurrent radiotherapy (PRR), post-recurrent transarterial chemoembolization (PRT), and post-recurrent percutaneous microwave coagulation (PRP). The tests were carried out in accordance with prognostic subtypes identified by machine learning algorithms.

## Methods

### Patients

The framework was retrospectively derived using a pooled dataset from patients with ICC who received surgical resection at the Eastern Hepatobiliary Surgery Hospital, Second Military Medical University (*n* = 1,477), Renji Hospital, School of Medicine, Shanghai Jiao Tong University (*n* = 106), and Mengchao Hepatobiliary Hospital, Fujian Medical University (*n* = 14) between 2008 and 2015, which was independently validated by the patients from Zhongshan Hospital, Fudan University (*n* = 246). All four databases satisfied the following inclusion criteria: Eastern Cooperative Oncology Group (ECOG) performance status of 0–1, no neoadjuvant treatment, no mixed hepatocellular-cholangiocarcinoma and hilar/distal cholangiocarcinoma, no perioperative death (within 30 days after surgery), and no distant metastasis. In the quality assessment, 188 patients were excluded due to incomplete data, and thus a total of 1,421 and 234 patients were finally enrolled for the model training and validation, respectively. This study was carried out in accordance with the TRIPOD statement. The protocol was approved by the Ethics Committee of Renji Hospital, School of Medicine, Shanghai Jiao Tong University. All subjects gave written informed consent in accordance with the Declaration of Helsinki.

### Diagnosis

Diagnosis of ICC was performed based on results of clinical, serological, and imaging studies (contrast-enhanced computed tomography [CT] and/or magnetic resonance imaging [MRI]). Positron emission tomography (PET) was performed in patients suspicious of metastases according to clinical and radiological characteristics. After surgery, CK7, CK19, and MUC1 positivity along with CK20, HepPar1, and glypican-3 negativity was considered pathological confirmation of ICC (18).

### Clinical Interventions

Resection was carried out according to the size and location of tumor, estimated post-operative liver volume, and the Couinaud segmentation as described before (16). Hepatoduodenal ligament, retropancreatic, and paraaortic lymph nodes were routinely dissected. Perihepatic lymph node metastasis identified by preoperative CT/PET was considered for surgery if considered completely removable.

PAT was considered after discussion of the pros and cons of PAT by the operating surgeons and patients. The performance of PAT mostly depended on their socioeconomic status and intention. Among the patients who actively agreed to receive PAT, those with Child-Pugh class of A to B, ECOG score of 0–1, normal kidney function, no evidence of extrahepatic metastasis, platelet count above 50 × 10^9^/L, and white blood cell count above 3 × 10^9^/L were enrolled. PAT was performed within 2 months after resection by injecting 3–5 ml of iodized oil emulsion (Lipiodol, Guerbet Laboratories) with 500 mg of 5-fluorouracil (FU), 10 mg of hydroxycamptothecin, and 20 mg of epirubicin (19).

For recurrent ICC, gemcitabine and/or 5-FU-based PRC was prior for metastatic diseases, whereas a dose volume histogram-based PRR (90% of dose curve covered by the plan target volume) was preferentially performed in patients with large tumors and/or vascular invasion; there was no priority between PRT and PRR, in line with the National Comprehensive Cancer Network (NCCN) guidelines. PRT was carried out using the same methodology as PAT. PRP was proceeded using an MTC-3 microwave generator (2,450 MHz, 1–100 W) at 80–100 W for 3–5 min automatically with a safety margin of 1 cm (20). Supportive information related to inclusion of patients for standardized performance of the procedures is described in the Supplementary Material.

### Follow-up

Active follow-up of serum carbohydrate antigen (CA) 19-9, carcinoembryonic antigen (CEA), alpha fetoprotein (AFP), liver function tests, and the abdominal ultrasound was made by patients once per 2 months within 2 years after surgery and once per 3–6 months thereafter. Patients without active visits were contacted by telephone inquiries. CT/MRI was performed once per 6 months or less when recurrence was suspicious. Development of new lesions with radiological characteristics of ICC was considered as a recurrence. Follow-up was discontinued at the time of death. The terms “disease-free survival (DFS)” and “overall survival (OS)” were defined as time from surgery to the detection of recurrence/metastasis and death, respectively.

### Network Architecture and Derivation Procedures

The authors who derived the framework were blinded to the validation dataset, whereas those who validated the framework were blinded to the derivation dataset. To infer an estimated probability for latent risk and latent stable as output, we conducted a 12 × 1 vectors, including 12 clinical indicators, in the input layer via full-connected hidden layers (12 × 28, 28 × 28, 28 × 14, and 14 × 28 nodes). For the given hidden layer *i*, we applied tanh for activation function between input *x* and output *y*:

$$\begin{array}{l} \;y=f_{i}\left(x\right)=\tanh\left(W_{i}x\;+\;b_{i}\right)\\ \tanh x=\;\frac{sinhx}{coshx}=\;\frac{e^{x}-e^{-x}}{e^{x}+e^{-x}} \end{array}$$

*x* and *y* are two arrays of the sized p and q, whereas *W*_*i*_ and *b*_*i*_ are the weight matrix and the intercept array, respectively. For the output layer, we used the softmax as an activation function:

$$\begin{array}{l} \;y=f_{o}\left(x\right)=softmax\left(W_{i}x\;+\;b_{i}\right)\\ softmax\;f_{i}\left(\vec{x}\right)=\frac{e^{x_{i}}}{\sum_{j=1}^{J}e^{x_{j}}}\;for\;i=1,\;\ldots ,\;J \end{array}$$

For the neural network with *k* layers, *y* is driven from:

$$\begin{array}{l} y=F_{1\to k}\left(x\right)=\;{f_{k}}^{\circ }f_{k-1}\ldots^{\circ }f_{1}\left(x\right) \end{array}$$

where ${f_{k}}^{\circ }f_{k-1}\left(x\right)=\;f_{k}\left(f_{k-1}(x)\right)$ is the composed function of *f*_*k*_ with *f*_*k*−1_. To train this AI framework to find the different weight vectors *W*_*i*_ and bias *b*_*i*_ by minimizing the error between predicted output and actual class, we chose cross entropy as the loss function, which indicates the error between predicted *y*_*pred*_ and actual ending *y*_*actual*_.

$$\begin{array}{l} Cross\;entropy\;H\left(y_{actual},y_{pred}\right)=-\sum y_{actual}{\left(x\right)}^{*}log{\;y}_{pred}\left(x\right) \end{array}$$

*W*_*i*_ and *b*_*i*_ were initialized with truncated normal distribution (standard deviation = 0.1; https://www.tensorflow.org/api_docs/python/tf/truncated_normal). The Adam Optimizer algorithm (initial learning rate=0.001) was used to minimize the loss function via backpropagation to update weights and biases per layer (21). In addition, we have applied a dropout layer by randomly dropping 30% weights before the output layer to improve the generalization ability, but application of the weight decay was found to decrease the performance ability of the AI framework. The model was trained for 1,500 iterations with a batch size 200 in producing a model update to support multiple updates for each iteration.

### Definition of the Prognostic Subtypes

The term “latent risk (AI-framework-estimated recurrence probability > 0.5)” refers to a subset of ICCs that are under severe risk of recurrence at any time after resection; resection of the tumor is therefore not likely to be curative regardless of curative intent. “Latent stable (AI-framework-estimated recurrence probability <0.5)” refers to a relatively constant disease status that resection of the tumor provides a long-term satisfactory prognosis. To support understanding, latent risk and latent stable can be simply considered as AI-high risk and AI-low risk, respectively.

### Statistical Analysis

The primary and secondary endpoints were DFS and OS, respectively. The model was evaluated by comparing with the AJCC stage and Cox multivariate hazard proportional model-derived individualized scores, which were indicated by changes in χ^2^, integrated discrimination improvement (IDI) and a net reclassification improvement (NRI) with 95% confidence interval (CI), and receiver operating characteristic (ROC) curves with area under curve (AUC) values. Although an AJCC stage for ICC was not developed with intent for survival prediction, it still is the most commonly applied staging system in clinical medicine supportive of survival estimation. Kaplan-Meier (KM) curves with the log-rank test for *P*-value and Mantel-Haenszel for hazard ratio (HR) were generated for evaluation and digitalization of survival outcomes. *P* < 0.05 is regarded statistically significant. The ICC AI framework was constructed using the TensorFlow (v1.2.1) on servers equipped with dual Intel (R) Core (TM) i7-4650U CPU @1.70 Ghz 2.30 GHz, 8 GB RAM, and Intel (R) HD Graphics 5000. All statistical analyses were performed using Python (v3.6.5) and R Project for Statistical Computing (v3.4.4).

## Results

### Development of the ICC AI Framework

**Figure 1:**
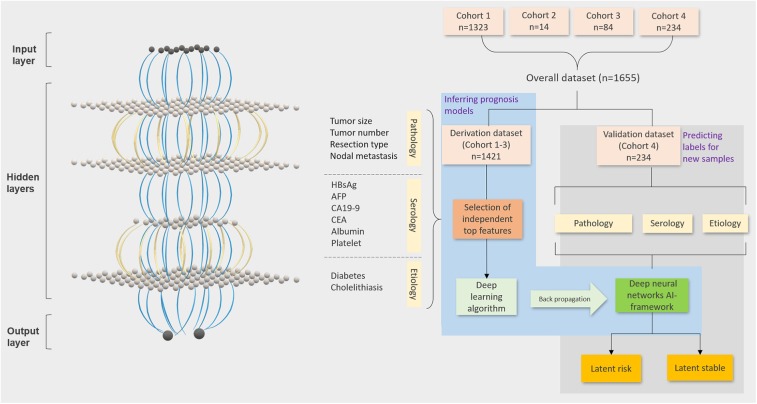
Workflow of the ICC AI-framework. TensorFlow-based deep learning and machine learning techniques to evaluate latent risk ICC by integrating the generally obtainable pathologic, serologic, and etiologic clinical factors of the patients from four independent clinical centers. The workflow includes four steps (Step 1: randomization of derivation and validation datasets; Step 2: Selection of the significant covariates; Step 3: deep learning algorithm for evaluation of individual scores; Step 4: stratification of latent risk and stable).

An AI framework to evaluate individualized probabilities for identifying categorical prognostic subtypes was developed. For this purpose, independent significant covariate features and the DL algorithm were selected using non-overlapping derivation and validation datasets (Figure 1). Demographic, etiological, pathological, and serological characteristics were evaluated using univariable and multivariable Cox regression models for disease-free survival. The 28 evaluated characteristics were gender; age; hepatitis B virus (HBV) and hepatitis C virus infections; HBV DNA; antiviral treatment; syphilis infection; liver cirrhosis and fluke; fatty liver; smoking and alcohol abuse; diabetes mellitus; hypertension; tumor location and differentiation; AFP, CA 19-9, 125, and 242; CEA; albumin; platelet count; vascular invasion; lymph node metastasis; tumor size and number; and surgical extent. The evaluation identified 12 of the features as most important, including tumor size and number, surgical extent, lymph node metastasis, hepatitis B surface antigen (HBsAg), AFP, CA19-9, CEA, albumin, platelet count, diabetes mellitus, and cholelithiasis (Tables 1, 2). Albumin (>35 vs. ≤ 35 g/L), AFP (>50 vs. ≤ 50 ng/ml), and CA 19-9 (>37 vs. ≤ 37 U/ml) were categorized into normal and abnormal groups according to the standardized cut-off values for normal ranges; the platelet count was stratified into <100, 100–300, and >300 × 10^9^/L; CEA was stratified into <2.5, 2.5–5.0, and >5.0 ng/ml; tumor size was stratified into <2.5, 2.5–5.0, and >5.0 cm; and tumor number was categorized into single, double, and multiple tumors. Tests were conducted to confirm if the covariates were significant prognostic factors for the OS in the derivation dataset. The multivariable analysis found all involved factors, except albumin and diabetes, to be significantly and independently predictive of the OS (Supplementary Table 1). Additionally, HBsAg, AFP, tumor size, and resection type were identified as insignificant independent prognostic factors in the validation dataset (Supplementary Table 2). Finally, a training dataset (*n* = 1,421) was used to derive the framework based on the 12 identified features. The framework was derived with time-to-event outcomes using a backpropagation technique, which synchronously updated each lay's weights and biases to optimize the statistical likelihood of the framework.

**Table 1:** Baseline demographic and clinical characteristics of the patients.

	**Derivation dataset** **(*n* = 1,421)**	**Validation dataset** **(*n* = 234)**
Age, years	57 (49–64)	58 (50–65)
Gender, male	915 (64.4)	140 (59.8)
Albumin, g/L	40.4 (36.1–43.5)	41.0 (39.0–43.0)
Platelet count, 10^9^/L	184 (142–238)	189 (147–228)
Diabetes	136 (9.6)	25 (10.7)
HBV infection, HBsAg	624 (43.9)	28 (12.0)
Cholelithiasis	132 (9.3)	18 (7.7)
AFP, ng/ml	3.0 (2.0–5.5)	2.8 (1.9–4.4)
CA19–9, U/ml	57.8 (17.8–548.1)	32.1 (11.6–239.0)
CEA, ng/ml	2.8 (1.7–5.7)	2.4 (1.5–4.8)
Tumor size, cm	6.0 (4.0–8.0)	5.0 (3.5–8.0)
Tumor number		
Single	1221 (85.9)	188 (80.3)
Multiple	200 (14.1)	46 (19.7)
Lymph node metastasis	332 (23.4)	60 (25.6)
Resection type		
Minor hepatectomy	1052 (74.0)	134 (57.3)
Hemi or extended hepatectomy	369 (26.0)	100 (42.7)
TNM stage^a^		
I–II	1089 (76.6)	174 (74.4)
III–IV	332 (23.4)	60 (25.6)

*Data are n (%) or median (IQR). HBsAg, hepatitis B surface antigen; AFP, alpha fetoprotein; CA, carbohydrate antigen; CEA, carcinoembryonic antigen*.

a *TNM stage: American Joint Committee on Cancer 8th edition staging for intrahepatic cholangiocarcinoma*.

**Table 2:** Selection of top covariates using the Cox multivariable regression.

	**Univariable analysis**		**Multivariable analysis**	
	**HR (95% CI)**	***P*-value**	**HR (95% CI)**	***P*-value**
Albumin <35 g/L	1.96 (1.66–2.31)	<0.001	1.26 (1.05–1.51)	0.015
Platelet count, × 10^9^/L^a^	1.68 (1.45–1.94)	<0.001	1.21 (1.04–1.40)	0.011
Diabetes	1.63 (1.34–1.99)	<0.001	1.41 (1.15–1.72)	0.001
HBsAg	0.82 (0.72–0.93)	0.002	0.79 (0.69–0.90)	0.001
Cholelithiasis	1.57 (1.28–1.92)	<0.001	1.40 (1.13–1.73)	0.002
AFP >50 ng/ml	1.49 (1.19–1.86)	0.001	1.60 (1.26–2.02)	<0.001
CA19–9 > 37 U/ml	1.49 (1.32–1.69)	<0.001	1.18 (1.03–1.37)	0.020
CEA, ng/ml^b^	1.37 (1.27–1.47)	<0.001	1.12 (1.03–1.22)	0.011
Tumor size, cm^c^	1.69 (1.56–1.84)	<0.001	1.59 (1.46–1.73)	<0.001
Tumor number^d^	1.51 (1.37–1.67)	<0.001	1.28 (1.15–1.42)	<0.001
Lymph node metastasis	1.93 (1.68–2.22)	<0.001	1.40 (1.21–1.63)	<0.001
Resection type^e^	1.57 (1.42–1.74)	<0.001	1.17 (1.05–1.31)	0.005

*HR, hazard ratio; CI, confidence interval; HBsAg, hepatitis B surface antigen; AFP, alpha fetoprotein; CA, carbohydrate antigen; CEA, carcinoembryonic antigen*.

a *was stratified into <100, 100–300, and >300*.

b *was stratified into <2.5, 2.5–5.0, and >5.0*.

c *was stratified into ≤ 2.0, 2.1–3.0, 3.1–5.0, and >5.0*.

d *was stratified into single, double, and multiple*.

e *was stratified into minor hepatectomy, hemihepatectomy, and extended hepatectomy*.

### Validation of the ICC AI-Framework

**Figure 2:**
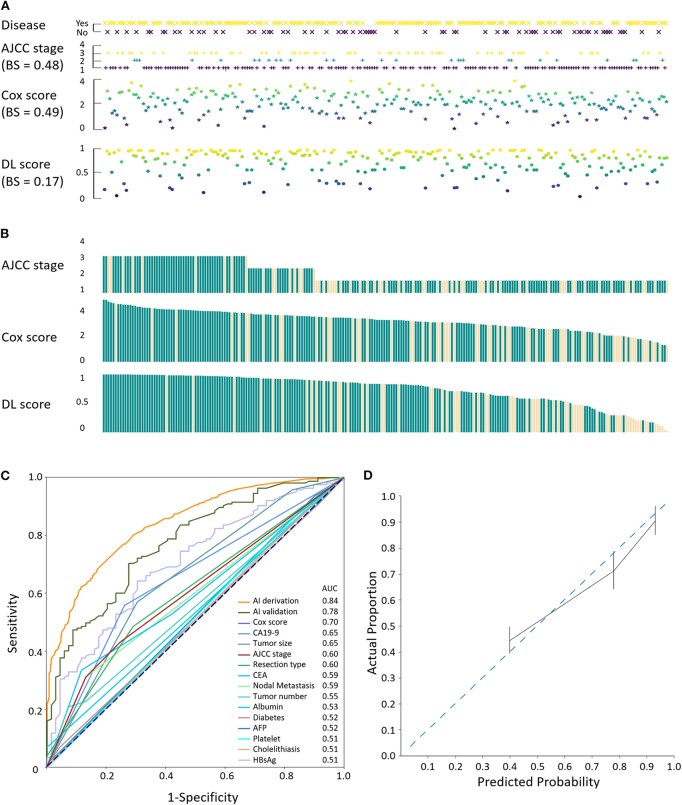
Validation of the ICC AI-framework. **(A)** Evaluation of the consistency between disease status and the AJCC stage, Cox score, and DL, respectively. BS, brier score. **(B)** Coherence comparison among staging/scoring systems. Light yellow, events. **(C)** ROC curves with AUC values of the AI derivation and validation, Cox score, AJCC stage, and involved covariates. **(D)** Calibration plot for evaluation of the actual proportion and predicted proportion of the events using the validation dataset.

The performance of the model was assessed by comparing the consistency of the disease status with that of the individualized stage/score from the validation set (*n* = 234; Figure 2A). Relative maldistributions were observed in the range-adjusted American Joint Committee on Cancer (AJCC) staging system (BS = 0.48) and the Cox multivariable models (BS = 0.49), whereas the framework (BS = 0.17) demonstrated well-propagated DL scores. Furthermore, visualization of the score-dependent disease status revealed predominance in the AI framework, demonstrating gradual increase of recurrence in proportion to the DL score (Figure 2B). Additionally, the AI framework, covariates, AJCC staging system, and Cox multivariable regression were evaluated using ROC curves and AUC values, and the Cox score and AJCC stage were evaluated by the validation dataset (Figure 2C). The framework was derived (AUC = 0.84) and validated (AUC = 0.78) to be excellent, whereas the AJCC stage (AUC = 0.60) and Cox score (AUC = 0.70) showed less sensitivity. Calibration plot also showed good association between actual proportion and predicted probability for the AI framework (Figure 2D). In terms of integrated discrimination index (IDI) and net reclassification index (NRI), performance of the framework was significantly better compared with the AJCC (derivation: χ^2^ = 54.93, *P* < 0.001, IDI = 0.30, NRI = 19.62; validation: χ^2^ = 7.22, *P* < 0.001, IDI = 0.29, NRI = 11.85) and Cox (derivation: χ^2^ = 849.09, *P* < 0.001, IDI = 0.51, NRI = 63.46; validation: χ^2^ = 146.44, *P* < 0.001, IDI = 0.46, NRI = 46.11) models (Table 3).

**Table 3:** Discriminative and risk: reclassification ability of the ICC AI-framework.

	**Model performance**		**IDI (95% CI)**	**Risk reclassification**				
	**Change in χ^2^**	***P*-value**		**Events**		**Non-events**		**NRI (95%CI)**
				**Risk up**	**Risk down**	**Risk up**	**Risk down**	
**AI vs. Cox**								
Derivation	849.09	<0.001	0.51 (0.50–0.52)	0.90	0.06	0.54	0.34	63.46 (61.68–65.24)
Validation	146.44	<0.001	0.46 (0.44–0.47)	0.88	0.08	0.61	0.29	46.11 (40.56–51.66)
**AI vs. AJCC**								
Derivation	54.929	<0.001	0.30 (0.29–0.30)	0.64	<0.01	0.48	0.03	19.62 (18.8–20.45)
Validation	7.2197	0.007	0.29 (0.28–0.31)	0.61	<0.01	0.54	0.04	11.85 (9.39–14.32)

*IDI, integrated discrimination improvement; CI, confidence interval; NRI, net reclassification improvement; AJCC, American Joint Committee on Cancer*.

### Survival Outcomes of the Latent Risk and Stable Subtypes

**Figure 3:**
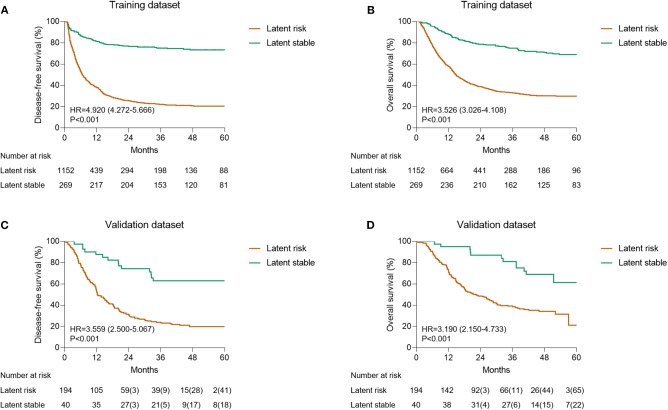
Kaplan-Meier estimation of the prognostic subtypes. **(A)** The OS of training dataset according to the latent status. **(B)** The DFS of training dataset according to latent status. **(C)** The OS of validation dataset according to the latent status. **(D)** The DFS of validation dataset according to the latent status.

Taking into consideration the entire dataset, the ratio of the latent risk group to the stable group was found to be ~8:2 using probabilistic stratification of the AI framework. KM curves were generated to evaluate the prognostic subtypes. The differences between latent risk and stable groups in disease-free survival (DFS) (HR, 4.920; 95% CI, 4.272–5.666; *P* < 0.001; Figure 3A) and overall survival (OS) (HR, 3.526; 95% CI, 3.026–4.108; *P* < 0.001; Figure 3B) in the training dataset were significant. On the contrary, in the validation dataset, similar results were observed in both DFS (HR, 3.559; 95% CI, 2.500–5.067; *P* < 0.001; Figure 3C) and OS (HR, 3.190; 95% CI, 2.150–4.733; *P* < 0.001; Figure 3D). The censored subjects-excluded 1-, 3-, and 5-year OS were 95.0, 79.4, and 38.9% vs. 73.2, 36.1, and 2.3%, respectively, in the latent stable group compared to latent risk group, and the DFS were 87.5, 60.0, and 36.4% vs. 54.1, 21.1, and 1.3%, respectively, in the validation dataset.

### Potential Applicability of the AI-Prognostic Subtypes

**Figure 4:**
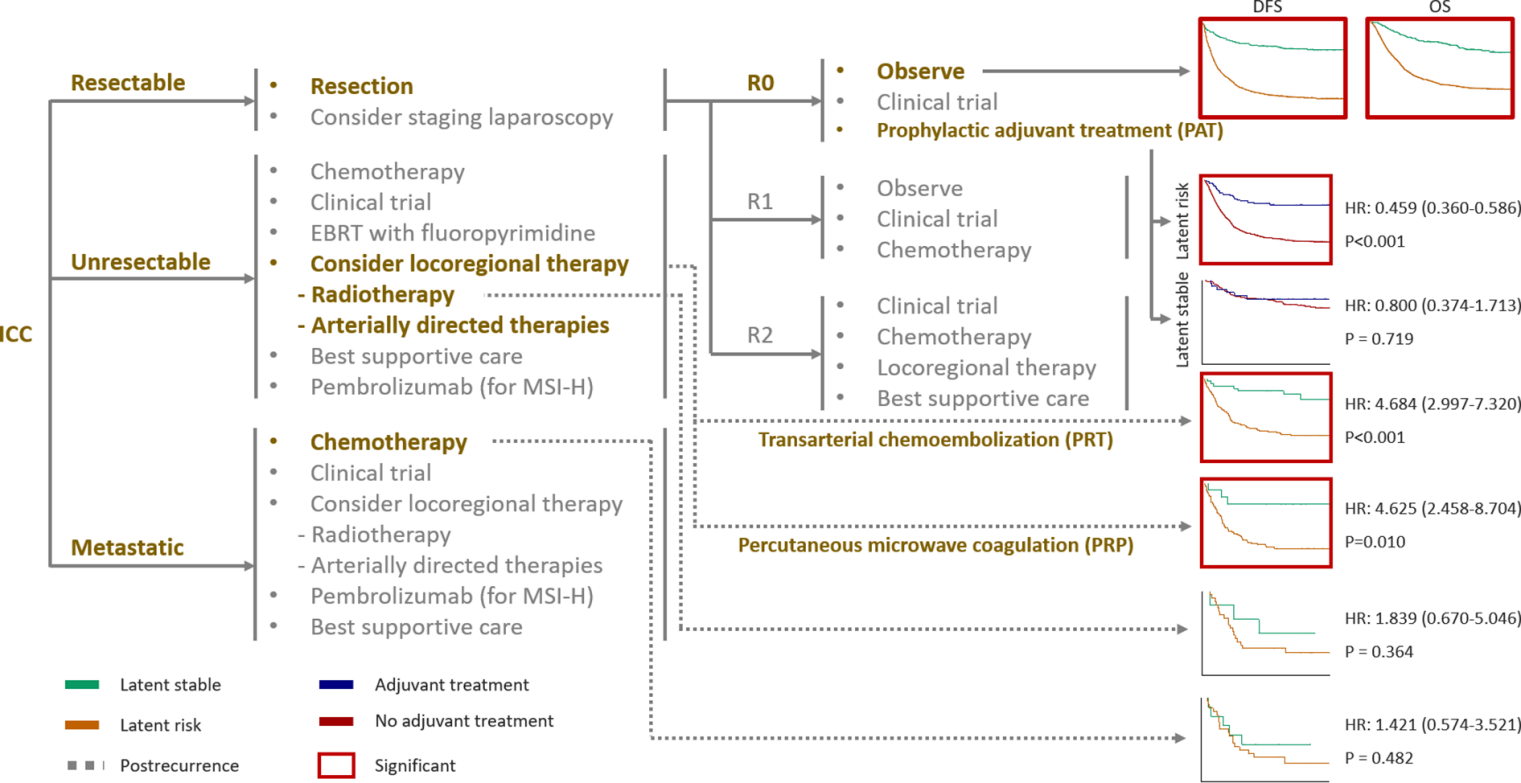
Outcomes of the NCCN guidelines clinical interventions according to the latent status. Kaplan-Meier curves were generated for each clinical intervention, including prophylactic adjuvant treatment and recurrence treatment, according to the latent status. For generation of the survival curves, post-recurrence survival was applied for transarterial chemoembolization, percutaneous microwave coagulation, radiotherapy, and chemotherapy, whereas overall survival was applied for prophylactic adjuvant treatment.

In this paper, an attempt has been made to study whether an AI framework is able to provide guidance for clinical interventions as recommended in NCCN as seen in Figure 4 (22). While evaluating the effectiveness, PAT can result into significant survival benefit (median survival benefit, 19 months; HR, 0.459; 95% CI, 0.360–0.586; *P* < 0.001) in the latent risk group. However, no significant difference was observed in the latent stable group (HR, 0.800; 95% CI, 0.374–1.713; *P* = 0.719). In case of the local intrahepatic recurrent patients, the AI-framework-derived prognostic subtypes could be effectively utilized to stratify patients who have been significantly benefited from PRT (HR, 4.684; 95% CI, 2.997–7.320; *P* < 0.001) and PRP (HR, 4.625; 95% CI, 2.458–8.704; *P* < 0.001), respectively. On the contrary, the patients who underwent radiotherapy did not show any significant difference as seen in case of the latent risk and stable groups (HR, 1.839; 95% CI, 0.670–5.046; *P* = 0.364). Moreover, chemotherapy did not indicate any significant results of survival amongst the prognostic subtypes (HR, 1.421; 95% CI, 0.574–3.521; *P* = 0.482).

## Discussion

We adopted a DL approach to learn prognostic prediction using significant clinical factors and created dimidiate prognostic subtypes with distinctive prognosis and efficacy of clinical interventions. This model was compared for accuracy with the most widely used, pre-existing AJCC staging system and the Cox methodology, which was systematically evaluated in context to current clinical standard for recurrent ICC. In comparison to the previous studies on prediction of OS, the current framework specifically caters to cancer-specific survival, excluding mortality due to unknown causes. Moreover, this approach increases the accuracy of equal covariates-generated Cox multivariable hazard proportional model and the stratified prognostic subtypes depicting significant differences amongst various recurring treatments. Collectively, the DL approach was found to be effective in estimation of survival and to devise a categorical strategy to deal with heterogeneity of ICCs by classifying them into latent risk and stable groups for clinical interventions.

We have attempted to maximize the chances for identification of prognostic factors for ICC since it is a disease with diverse outcomes and the issues in identification of the prognostic factors arises due to its exclusivity (23, 24). Therefore, we adopted an 8:2 ratio in randomization of the derivation and validation datasets for meticulous detection of prognostic factors, which enabled us to detect 12 independent prognostic factors. These factors are pre-specified by the Cox hazards regression model, as it is difficult to apply different factors owing to complexity.

Recent studies have demonstrated that a post-operative prophylactic adjuvant therapeutic approach can account for significant survival benefits by preventing events or by prolonging the time-to-recurrence (25–27). Latent risk ICC might be the reason for survival benefits, because the latent stable group was associated with significantly favorable prognosis without the application of adjuvant treatments.

According to the National Cancer Database of the American College of Surgeons and the American Cancer Society that takes into account ~70% of the US population, the median OS for chemotherapy (*n* = 2,176) and chemoradiotherapy (*n* = 666) are 10.5 (95% CI, 10.0–11.5) and 13.6 (95% CI, 12.3–15.7) months, respectively, for unresectable ICC (28, 29). In our study, estimated latent risk for recurrent ICC treated with chemotherapy and chemoradiotherapy showed no significance compared with the latent stable. Recent studies have suggested, with reasonable evidence, that the application concurrent chemoradiotherapy has better efficacy is better than solely applying adjuvant chemotherapy or radiotherapy (28, 29). Therefore, evaluation of the latent risk combined with chemoradiotherapy may lead to significant survival benefits, though this awaits further validation by future trials.

Although our study provides insights into the use of DL for ICC in a clinical factor setting, some clinical interventions, such as immunotherapy and liver transplantation, are not involved, and the framework is therefore not comprehensive for all circumstances. Furthermore, while we have used our techniques for ICC—the application of the AI-based clinical factors-derived estimative approaches for other tumors might provide auxiliary perspicacious insights. The evaluation of the resection candidate also needs to be considered. There were few patients with regional disease, which is considered not a candidate for surgical resection in some surgery centers. Lastly, the proportion of etiologic subtypes needs to be considered when interpreting the results. There were relatively large proportion of ICCs arose from HBV infection, which is not prevalent in Western countries. Therefore, validation by Western population is essential for the framework to be applied in clinical practice.

In conclusion, the AI approach revealed precision prognostic estimation compared to the AJCC stage for ICC and Cox multivariable regression model in terms of survival prediction and prognostic subtype stratification in patients with ICC after resection. Future validation studies are required to confirm its applicability in patients with ICC from other regions and in other cancers.

## Data Availability Statement

Datasets for this study are available from the involved authors under reasonable request.

## Ethics Statement

Ethical approval was not provided for this study on human participants because it was waived by the institutional review board according to retrospective nature of the study. The patients/participants provided their written informed consent.

## Author Contributions

SJ, YG, JC, QG, JL, HW, QX, and LC designed the study. SJ, YG, JC, QG, GL, BZ, MS, FS, QC, CS, JL, HW, QX, and LC collected data. SJ, YG, JC, QG, JL, HW, QX, and LC performed analyses. SJ, YG, JC, QG, GL, BZ, MS, FS, QC, CS, JL, HW, QX, and LC were involved in data interpretation. All authors drafted, reviewed, and approved the manuscript.

### Conflict of Interest

The authors declare that the research was conducted in the absence of any commercial or financial relationships that could be construed as a potential conflict of interest.
