# Supplementary material for: Latent Risk Intrahepatic Cholangiocarcinoma Susceptible to Adjuvant Treatment After Resection: A Clinical Deep Learning Approach
Source: Front Oncol. 2020 Feb 19;10:143. doi: 10.3389/fonc.2020.00143 (PMC7042372; doi:10.3389/fonc.2020.00143)
Supplement: Supplementary file 1 [file Table_1.docx]

**Supportive information related to inclusion of patients for standardized performance of the procedures**

This study is a retrospective multicenter real-world study. Inclusion and exclusion criteria were standardized among all involved hospitals. All patients received hepatic resection for ICC, and recurrence after surgical resection is the primary endpoint of this study. The presents study did not involve any portal vein embolization+resection or associating liver partition and portal vein ligation for staged hepatectomy (ALPPS). Only the patients with hepatic resection as initial treatment were involved. Generally, patients with unresectable locoregional recurrence without extrahepatic metastasis received locoregional therapy, including transarterial chemoembolization, percutaneous microwave coagulation, and radiotherapy. The selection between TACE and PMCT was based on tumor size that relatively large tumors (>3 cm) received TACE and relatively small tumors received PMCT, and others who were not eligible for TACE (due to tolerability) or PMCT (due to tumor size) received radiotherapy. Furthermore, those with vascular invasion (portal vein) at the time of recurrence also received radiotherapy. In addition, patients with recurrence with extrahepatic metastasis received chemotherapy. In addition to the above terms, the following criteria also had to be satisfactory for patients to receive locoregional therapy, including TACE and PMCT: (1) Child-Pugh class A or B, (B) white blood cell count≥3.0 × 10^9^/L, (C) platelet count≥50 × 10^9^/L, (D)normal kidney function, (E) no evidence of extrahepatic metastasis, and (F) Eastern Cooperative Oncology Group score of 0 to 1.

**Supplementary Table 1. Univariable and multivariable analyses of the significant covariates for the overall survival in derivation dataset**

|  | Univariable analysis | | Multivariable analysis | |
| --- | --- | --- | --- | --- |
|  | HR (95% CI) | P value | HR (95% CI) | P value |
| Albumin<35 g/L | 1.65 (1.37-1.97) | <0.001 | 1.07 (0.88-1.31) | 0.485 |
| Platelet count, ×10^9^/L^a^ | 1.71 (1.47-1.99) | <0.001 | 1.22 (1.04-1.43) | 0.014 |
| Diabetes | 1.25 (1.01-1.56) | 0.042 | 1.00 (0.80-1.25) | 0.997 |
| HBsAg | 0.82 (0.71-0.94) | 0.004 | 0.82 (0.71-0.95) | 0.006 |
| Cholelithiasis | 1.64 (1.33-2.02) | <0.001 | 1.47 (1.18-1.83) | 0.001 |
| AFP>50 ng/ml | 1.37 (1.09-1.73) | 0.008 | 1.30 (1.02-1.65) | 0.035 |
| CA19-9>37 U/ml | 1.48 (1.29-1.69) | <0.001 | 1.26 (1.08-1.46) | 0.003 |
| CEA, ng/ml^b^ | 1.30 (1.20-1.41) | <0.001 | 1.08 (0.98-1.18) | 0.111 |
| Tumor size, cm^c^ | 1.54 (1.41-1.68) | <0.001 | 1.45 (1.33-1.58) | <0.001 |
| Tumor number^d^ | 1.40 (1.26-1.55) | <0.001 | 1.18 (1.06-1.32) | 0.003 |
| Lymph node metastasis | 1.69 (1.46-1.96) | <0.001 | 1.21 (1.03-1.42) | 0.020 |
| Resection type^e^ | 1.51 (1.36-1.68) | <0.001 | 1.15 (1.02-1.29) | 0.023 |

HR, hazard ratio. CI, confidence interval. HBsAg, hepatitis B surface antigen. AFP, alpha fetoprotein. CA, carbohydrate antigen. CEA, carcinoembryonic antigen. ^a^was stratified into<100, 100-300, and>300. ^b^was stratified into<2.5, 2.5-5.0, and>5.0. ^c^was stratified into≤2.0, 2.1-3.0, 3.1-5.0, and>5.0. ^d^was stratified into single, double, and multiple. ^e^was stratified into minor hepatectomy, hemihepatectomy, and extanded hepatectomy.

**Supplementary Table 2. Univariable and multivariable analyses of the significant covariates for the overall survival in validation dataset**

|  | Univariable analysis | | Multivariable analysis | |
| --- | --- | --- | --- | --- |
|  | HR (95% CI) | P value | HR (95% CI) | P value |
| Albumin<35 g/L | 4.29 (2.29-8.05) | <0.001 | 2.14 (0.99-4.65) | 0.054 |
| Platelet count, ×10^9^/L^a^ | 2.73 (1.70-4.39) | <0.001 | 2.35 (1.39-3.99) | 0.002 |
| Diabetes | 1.50 (0.91-2.47) | 0.109 |  |  |
| HBsAg | 0.62 (0.35-1.09) | 0.094 |  |  |
| Cholelithiasis | 3.69 (2.19-6.21) | <0.001 | 2.20 (1.24-3.88) | 0.007 |
| AFP>50 ng/ml | 1.96 (1.12-3.42) | 0.018 | 1.47 (0.80-2.70) | 0.211 |
| CA19-9>37 U/ml | 2.68 (1.90-3.80) | <0.001 | 1.99 (1.37-2.90) | <0.001 |
| CEA, ng/ml^b^ | 1.77 (1.45-2.17) | <0.001 | 1.34 (1.08-1.67) | 0.009 |
| Tumor size, cm^c^ | 1.56 (1.27-1.91) | <0.001 | 1.24 (0.99-1.55) | 0.066 |
| Tumor number^d^ | 2.15 (1.77-2.60) | <0.001 | 1.75 (1.39-2.20) | <0.001 |
| Lymph node metastasis | 3.88 (2.72-5.53) | <0.001 | 2.05 (1.33-3.17) | 0.001 |
| Resection type^e^ | 1.64 (1.23-2.19) | 0.001 | 0.89 (0.63-1.25) | 0.491 |

HR, hazard ratio. CI, confidence interval. HBsAg, hepatitis B surface antigen. AFP, alpha fetoprotein. CA, carbohydrate antigen. CEA, carcinoembryonic antigen. ^a^was stratified into<100, 100-300, and>300. ^b^was stratified into<2.5, 2.5-5.0, and>5.0. ^c^was stratified into≤2.0, 2.1-3.0, 3.1-5.0, and>5.0. ^d^was stratified into single, double, and multiple. ^e^was stratified into minor hepatectomy, hemihepatectomy, and extanded hepatectomy.
